# Tonsillar cytokine expression between patients with tonsillar hypertrophy and recurrent tonsillitis

**DOI:** 10.1186/s13601-018-0205-z

**Published:** 2018-05-22

**Authors:** Emilia Mikola, Varpu Elenius, Maria Saarinen, Oscar Palomares, Matti Waris, Riitta Turunen, Tuomo Puhakka, Lotta Ivaska, Beate Rückert, Alar Aab, Tero Vahlberg, Tytti Vuorinen, Tobias Allander, Carlos A. Camargo, Mübeccel Akdis, Cezmi A. Akdis, Tuomas Jartti

**Affiliations:** 1 Department of Otorhinolaryngology, Turku University Hospital and Turku University, Turku, Finland; 2 Department of Paediatrics and Adolescent Medicine, Turku University Hospital and Turku University, P.O. Box 52, 20520 Turku, Finland; 3 Swiss Institute of Allergy and Asthma Research, University of Zürich, Davos, Switzerland; 4 Christine Kühne-Center for Allergy Research and Education, Davos, Switzerland; 5 Department of Biochemistry and Molecular Biology, School of Chemistry, Complutense University of Madrid, Madrid, Spain; 6 Department of Clinical Virology, Turku University Hospital, Turku, Finland; 7 Department of Virology, University of Turku, Turku, Finland; 8 Department of Otorhinolaryngology, Satakunta Central Hospital, Pori, Finland; 9 Department of Biostatistics, University of Turku and Turku University Hospital, Turku, Finland; 10 Department of Clinical Microbiology, Karolinska University Hospital, Stockholm, Sweden; 11 Department of Emergency Medicine, Massachusetts General Hospital, Harvard Medical School, Boston, USA; 12 Division of Rheumatology, Allergy and Immunology, Department of Medicine, Massachusetts General Hospital, Harvard Medical School, Boston, USA

**Keywords:** Allergy, Asthma, Child, Cytokine, Interferon, Interleukin, T helper cell, Tonsil, Virus

## Abstract

**Background:**

Tonsils provide an innovative in vivo model for investigating immune response to infections and allergens. However, data are scarce on the differences in tonsillar virus infections and immune responses between patients with tonsillar hypertrophy or recurrent tonsillitis. We investigated the differences in virus detection and T cell and interferon gene expression in patients undergoing tonsillectomy due to tonsillar hypertrophy or recurrent tonsillitis.

**Methods:**

Tonsils of 89 surgical patients with tonsillar hypertrophy (n = 47) or recurrent tonsillitis (n = 42) were analysed. Patients were carefully characterized clinically. Standard questionnaire was used to asses preceding and allergy symptoms. Respiratory viruses were analysed in tonsils and nasopharynx by PCR. Quantitative real-time PCR was used to analyse intratonsillar gene expressions of IFN-α, IFN-β, IFN-γ, IL-10, IL-13, IL-17, IL-28, IL-29, IL-37, TGF-β, FOXP3, GATA3, RORC2 and Tbet.

**Results:**

Median age of the subjects was 15 years (range 2–60). Patients with tonsillar hypertrophy were younger, smoked less often, had less pollen allergy and had more adenovirus, bocavirus-1, coronavirus and rhinovirus in nasopharynx (all *P* < 0.05). Only bocavirus-1 was more often detected in hypertrophic tonsils (*P *< 0.05). In age-adjusted analysis, tonsillar hypertrophy was associated with higher mRNA expressions of IL-37 (*P* < 0.05).

**Conclusions:**

Intratonsillar T cell and interferon gene expressions appeared to be relatively stable for both tonsillar hypertrophy and recurrent tonsillitis. Of the studied cytokines, only newly discovered anti-inflammatory cytokine IL-37, was independently associated with tonsillar hypertrophy showing slightly stronger anti-inflammatory response in these patients.

**Electronic supplementary material:**

The online version of this article (10.1186/s13601-018-0205-z) contains supplementary material, which is available to authorized users.

## Background

Tonsillar disease is one of the most common disorders in the field of otorhinolaryngology. Different types of tonsillar disease include recurrent tonsillitis and tonsillar hypertrophy, with both leading to symptoms of mouth breathing, snoring, dyspnea, apnea or dysphagia. Treatment is usually antibiotics or tonsillectomy.

Tonsils are secondary lymphoid organs, which are centrally located at the beginning of the respiratory and gastrointestinal tracts where the immune system first comes into contact with infections agents and allergens [1]. Surgically removed palatine tonsils provide a conventional accessible source to study the interplay between foreign pathogens, allergens and the host immune system. Our previous studies demonstrated that tonsils are organs where immune regulation takes place in which allergen-specific regulatory T cells can be generated by mechanisms depending on plasmacytoid dendritic cells [2, 3]. The expression of T cell- and interferon specific genes in tonsils has been shown to be closely related to existing viral infections, age, and allergic illnesses [4, 5]. However, data are scarce on the differences in tonsillar virus infections and immune responses between patients with tonsillar hypertrophy or recurrent tonsillitis [6–8].

Tonsils appear to provide a good in vivo model for investigating the mechanisms of inflammatory processes and infections in lymphoid organs [2–5]. Therefore, we studied whether virus detection and T cell and interferon gene expressions differed between the two main indications of surgery, tonsillar hypertrophy or recurrent tonsillitis.

## Methods

### Patients

Human tonsil samples used in this study were acquired from 200 consecutive tonsillectomy patients who underwent tonsillectomy in Satakunta Central Hospital, Pori, Finland between April 2008 and March 2009. The inclusion criteria were elective tonsillectomy according to clinical indication and written informed consent from the study patient and/or his/her guardian. The study protocol was approved by the ethics committee of Satakunta Central Hospital, Pori, Finland. Study was initiated only after obtaining written consent from the participant or his/her guardian.

### Study protocol and sample collection

A standard questionnaire was used to obtain information on allergic diseases and respiratory symptoms within 30 days before the operation (Additional file 1: Table 1). Tonsillectomy was performed according to routine clinical procedure. Internal tonsillar tissue was immediately cut in 3–4 mm cubes, stored in RNA*late*r RNA stabilization reagent (Qiagen, Hilden, Germany), incubated at + 4 °C until next working day and finally stored at − 80 °C after removal of the non-absorbed reagent. For viral analyses, a part of the tonsils and a nasopharyngeal aspirate were stored in dry tubes at − 80 °C [4]. Nasopharyngeal aspirate samples were obtained during anaesthesia using a standardized procedure [4]. Serum total 25(OH)D measurement was done using an immunoassay (Abbott Architect, Chicago, USA) and bioavailable levels of 25(OH)D were estimated using additional serum measurements (D-binding protein and albumin) and published formulae.

### Definitions

Tonsillar hypertrophy group was defined as patients who underwent tonsillectomy because of obstructive symptoms such as snoring, breathing difficulties or swallowing problems. There were no tonsillar infection problems in this group. Recurrent tonsillitis group was defined as patients who underwent tonsillectomy because of recurrently infected tonsils (viral or bacterial) during the past 6–12 months. Those operated because of acute infection or peritonsillar abscess were excluded.

### Analysis of viruses and cytokines

In-house real-time PCR assays were used to detect human bocavirus-1, rhinovirus, enterovirus, and respiratory syncytial virus as described previously [4]. Seeplex RV12 ACE Detection (Seegene, Seoul, Korea) multiplex PCR assay was used for detection of adenovirus, coronaviruses (229E/NL63 and OC43/HKU1), influenza A and B viruses, metapneumovirus, parainfluenza virus types 1-3, respiratory syncytial virus group A and B, and rhinovirus [4, 5]. Virus diagnostics were carried out in the Department of Virology, University of Turku, Turku, Finland, and in the Department of Clinical Microbiology, Karolinska University Hospital, Stockholm, Sweden.

To isolate total RNA from palatine tonsils, tissues (previously stabilized in RNA*later*) were homogenized in grinding tubes containing CK28 ceramic beads by using a Precellys 24 homogenizer (Bertin Technologies, Montigny le Bretonneux, France) two times at 6000 rpm for 50 s [4]. Total RNA from cell samples was isolated using the RNeasy mini kit (Qiagen, Hilden, Germany). Reverse transcription was performed with the Revert Aid M-MuLV Reverse Transcriptase (Fermentas, St. Leon-Rot, Germany) using random hexamer primers according to the manufacturers protocol. Gene expressions of IFN-α, IFN-β, IFN-γ, IL-10, IL-13, IL-17, IL-28, IL-29, IL-37, TGF-β, FOXP3, GATA3, RORC2 and Tbet were analysed by quantitative real-time PCR using iTaq SYBR Green Supermix with ROX (Bio-Rad, Hercules, CA, USA) on a 7900HT Fast Real-Time PCR instrument (Applied Biosystems, Foster City, CA, USA). Housekeeping gene elongation factor 1α (EF1α) was used for normalization. Data are shown as relative expressions, which show 2^−(ΔCT)^ values multiplied by 10^4^, where ΔCT corresponds to the difference between the CT value for the gene of interest and EF1α.

### Statistical analysis

Continuous variables are described as medians and interquartile ranges, and were analysed using Mann–Whitney U test due to skewed distribution. Categorical variables are expressed as frequencies and percentages, and were analysed using Chi square test or Fisher exact test. Clinical, viral and immunological differences between study groups were analysed using unadjusted and multivariable linear regression analysis. The adjustments for immunologic analyses were chosen using backward stepwise multivariable models that initially included clinical factors and virus infections which significantly differed between the groups (age, self-reported pollen allergy, self-smoking, both adenotomy and tonsillectomy performed, respiratory symptoms one month prior to the operation and bioavailable 25(OH)D level). The final model was adjusted only for age. Before regression analyses, cytokine and transcription factor values were log-transformed because of positively skewed distributions. The mean difference was computed for log-transformed values: a recurrently infected group minus hypertrophic group. Statistical analysis was completed using JMP version 12.0.1 software (SAS Institute Inc. Cary, NC, USA). A two-sided *P* < 0.05 was considered statistically significant.

## Results

### Study population

**Fig. 1:**
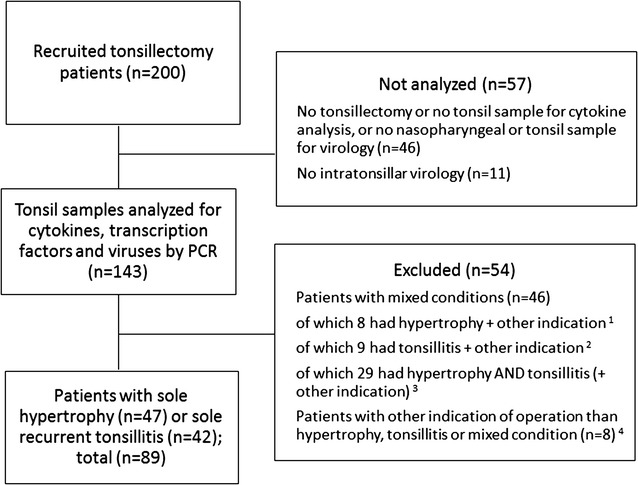
Study flow chart. ^1^8 had hypertrophy and another indication (recurrent otitis media n = 4; recurrent otitis media and fever n = 3; recurrent fever n = 1). ^2^9 had recurrent tonsillitis and another indication (recurrent fever n = 8; recurrent otitis media n = 1). ^3^21 had hypertrophy and tonsillitis; 8 had hypertrophy, tonsillitis, and another indication (recurrent fever, n = 5; recurrent otitis media, recurrent fever n = 1; recurrent otitis media n = 2). ^4^8 had other indication of operation than hypertrophy or tonsillitis (chronic white patches in tonsils n = 2; accumulation of food remnants, bad smelling breath, and feeling of beat in throat n = 1; recurrent fever n = 1; teeth braces n = 2; throat abscess n = 1; no clear cause n = 1)

Originally, 200 patients participated in the study. Of them, 46 subjects did not have remaining tonsil and/or nasopharyngeal samples for the current analysis, and 11 had no intratonsillar virology done in their samples. Another 54 subjects were excluded for having mixed indications of operation other than hypertrophy or tonsillitis (Fig. 1). Thus, 89 patients comprised the analytic cohort. Forty-seven (53%) of them had tonsillar hypertrophy and 42 (47%) had recurrent tonsillitis.

### Patient characteristics

All operations were performed during afebrile period of chronic tonsil condition. Respiratory symptoms on the operation day were present equally in the hypertrophy group and the recurrent tonsillitis group (15 vs. 18%, respectively; *P* = 0.72). The median age of the patients was 8 years (range 2–46) and 20 years (range 7–60), respectively (*P* < 0.001) (Table 1). In addition to being younger, patients in the hypertrophy group had more often adenotomy and tonsillectomy done, had less self-reported pollen allergy, smoked less, and had less throat pain, but had more often rhinitis and cough 1 month prior the operation and higher bioavailable 25(OH)D level than patients in the recurrent tonsillitis group (all *P* < 0.01) (Table 1). Otherwise no significant differences were found between the two groups.

**Table 1:** Patient characteristics

Characteristics	Tonsillar hypertrophy n = 47	Recurrent tonsillitis n = 42	*P* value
Age, years (range)	8 (2, 46)	20 (7, 60)	< *0.0001*
Male	27 (57%)	23 (55%)	0.80
Tonsillectomy and adenotomy	28 (60%)	5 (12%)	< *0.0001*
Self-reported allergy	20/43 (47%)	23/40 (58%)	0.32
Food	6/43 (14%)	3/39 (8%)	0.38
Drug	4/43 (9%)	4/39 (10%)	0.87
Seasonal, i.e. pollen	0/43 (0%)	7/39 (18%)	*0.004*
Perennial, i.e. animal or house dust mite	3/43 (7%)	2/39 (5%)	0.74
Other	2/43 (5%)	2/39 (5%)	0.90
Multiple	5/43 (12%)	4/39 (10%)	0.86
Physician-diagnosed atopic dermatitis	11/44 (25%)	4/38 (11%)	0.09
Self-reported allergic rhinitis	11/44 (25%)	11/39 (28%)	0.74
Physician-diagnosed asthma	6/42 (14%)	6/39 (15%)	0.89
Self-smoking	2/43 (5%)	14/40 (35%)	*0.0005*
Maternal smoking	15/44 (34%)	13/41 (32%)	0.82
Paternal smoking	16/41 (39%)	19/36 (53%)	0.23
Season of the surgery			0.85
Winter (months 12–2)	7 (15%)	4 (10%)	
Spring (months 3–5)	14 (30%)	13 (31%)	
Summer (months 6–8)	8 (17%)	5 (12%)	
Fall (months 9–11)	18 (38%)	20 (48%)	
Respiratory symptoms			
The operation day	6/39 (15%)	7/38 (18%)	0.72
Within 2 weeks	16/42 (38%)	12/37 (32%)	0.60
Within 4 weeks	23/42 (55%)	18/37 (48%)	0.59
One month prior the operation			
Throat pain	8/39 (21%)	14/35 (40%)	*0.0005*
Rhinitis	23/39 (59%)	6/35 (17%)	*0.0002*
Cough	15/39 (38%)	5/36 (14%)	*0.02*
Acute otitis media	2/39 (5%)	0/35 (0%)	0.17
Wheezing	2/39 (5%)	0/35 (0%)	0.17
25(OH)D level			
Total (nmol/l)	56.2 (41.7, 66.9)	45.7 (34.4, 72.3)	0.08
Free (pg/ml)	6.4 (4.9, 8.1)	5.0 (3.3, 8.8)	0.14
Bioavailable (ng/ml)	2.2 (1.7, 2.9)	1.6 (1.1, 2.7)	*0.01*

Values are shown as median (interquartile range, except age) or number of subjects (%). Data were analysed by Mann–Whitney U test, Chi square test, or Fisher’s Exact test

Significant values are shown in italic

### Viruses detected in nasopharyngeal aspirates and tonsils

Significantly more patients in the hypertrophy group, compared to the recurrent tonsillitis group, had a virus in their nasopharyngeal aspirates (79 vs. 38%, respectively; *P* **<**0.001). In addition, patients in the hypertrophy group had more often adenovirus, bocavirus-1, coronavirus or rhinovirus in nasopharyngeal aspirate (all *P *< 0.05) (Table 2). However, intratonsillar virus detection didn’t show statistically significant differences, except for bocavirus-1 which was detected in tonsils in 15% of patients with hypertrophy and only 2% of patients in recurrent tonsillitis group (Table 2). Patients in the hypertrophy group were more often positive for one virus in their nasopharyngeal aspirates (49 vs. 24%) or two viruses in their tonsils (11 vs. 0%, respectively) (both *P *< 0.05) (Table 2).

**Table 2:** Nasopharyngeal and intratonsillar virus detection

Virus	Nasopharynx		*P* value	Tonsil		*P* value
	Tonsillar hypertrophy n = 47	Recurrent tonsillitis n = 42		Tonsillar hypertrophy n = 47	Recurrent tonsillitis n = 42	
Adenovirus	9 (19%)	2 (5%)	*0.03*	7 (15%)	2 (5%)	0.10
Bocavirus-1	12 (26%)	2 (5%)	*0.005*	7 (15%)	1 (2%)	*0.03*
Coronavirus	3 (6%)	0 (0%)	*0.048*	0 (0%)	0 (0%)	–
Enteroviruses	4 (9%)	4 (10%)	0.87	6 (13%)	4 (10%)	0.63
Influenza A or B virus	1 (2%)	0 (0%)	0.26	0 (0%)	0 (0%)	–
Metapneumovirus	0 (0%)	1 (2%)	0.29	1 (2%)	0 (0%)	0.26
Parainfluenza virus types 1-4	1 (2%)	1 (2%)	0.94	4 (9%)	1 (2%)	0.19
Respiratory syncytial virus	1 (2%)	0 (0%)	0.26	2 (4%)	0 (0%)	0.11
Rhinovirus species A, B or C	27 (57%)	14 (33%)	*0.02*	2 (4%)	2 (5%)	0.91
Number of positive viruses	37 (79%)	16 (38%)	< *0.0001*	17 (36%)	8 (19%)	0.07
Positive for 1 virus	23 (49%)	10 (24%)	*0.01*	9 (19%)	7 (17%)	0.76
Positive for 2 viruses	8 (17%)	4 (10%)	0.30	5 (11%)	0 (0%)	*0.01*
Positive for 3 viruses	5 (11%)	2 (5%)	0.30	2 (4%)	1 (2%)	0.62
Positive for 4 viruses	1 (2%)	0 (0%)	0.26	1 (2%)	0 (0%)	0.26
Positive for ≥ 1 viruses	37 (79%)	16 (38%)		17 (36%)	8 (19%)	
Positive for ≥ 2 viruses	14 (30%)	6 (14%)		8 (17%)	1 (2%)	
Positive for ≥ 3 viruses	6 (13%)	2 (5%)		3 (7%)	1 (2%)	
Positive for ≥ 4 viruses	1 (2%)	0		1 (2%)	0	

Values are shown as number of subjects (%). Data were analysed by Chi square test, or Fisher’s Exact test

Significant values are shown in italic

### Cytokine and transcription factor expression profiles in tonsils

**Fig. 2:**
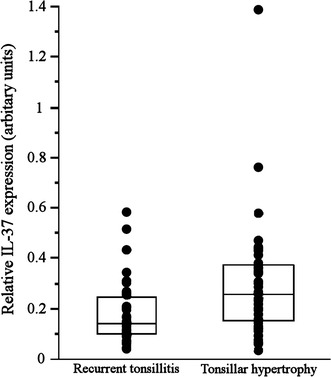
Relative tonsillar expression of IL-37. Forty-two recurrent tonsillitis samples compared with 47 hypertrophic tonsil samples. Values are arbitrary units x 10^4^ relative to EF1α. Data are represented as median with interquartile range. IL-37, Interleukin 37

In unadjusted analysis, patients in the hypertrophy group had stronger tonsillar expression of Tbet (*P* = 0.03) and IL-37 (*P* = 0.001) than patients in the recurrent tonsillitis group (Tables 3, 4). In the multivariable regression analysis, only age remained as a significant co-factor (Table 4). After adjustment for age, the expressions of only IL-37 was independently associated with tonsillar hypertrophy group (*P* < 0.05, Fig. 2). No other differences in cytokine or transcription factor expression were found between the groups.

**Table 3:** Intratonsillar cytokine and transcription factor expression in hypertrophic tonsils and recurrent tonsillitis

Cytokine or transcription factor	Tonsillar hypertrophy n = 47	Recurrent tonsillitis n = 42
	Median (IQR)	Median (IQR)
T-helper_1_		
IFN-γ	58 (37, 90)	76 (32, 117)
Tbet	54 (31, 83)	34 (22, 60)
T-helper_2_		
IL-13	1.2 (0.03, 3.7)	0.45 (0.02, 2.2)
GATA3	27 (16, 36)	18 (12, 39)
T-helper_17_		
IL-17	10 (5.6, 19)	7.3 (4.1, 13)
RORC2	15 (7.2, 31)	20 (10, 28)
T-regulatory		
IL-10	49 (24, 74)	35 (21, 64)
IL-37	0.26 (0.15, 0.37)	0.14 (0.10, 0.24)
FOXP3	46 (20, 87)	48 (24, 80)
TGF-β	163 (105, 232)	171 (120, 225)
Type I/III interferons		
IFN-α	15 (0.59, 62)	9.7 (0.27, 56)
IFN-β	24 (2.7, 101)	22 (2.1, 103)
IL-28	31 (3.1, 88)	12 (1.4, 75)
IL-29	11 (2.3, 34)	3.7 (1.3, 26)

Values are arbitrary units × 10^4^ relative to EF1α

IQR, interquartile range; IFN, interferon; Tbet, T-box transcription factor; IL, interleukin; GATA3, GATA-binding factor 3; RORC, RAR-related orphan receptor C; FOXP, forkhead box protein; TGF, tumour growth factor

**Table 4:** Differences in cytokine and transcription factor expression between hypertrophic tonsils and recurrent tonsillitis

Cytokine or transcription factor	Mean differences recurrently infected minus hypertrophic group			
	Univariate		Multivariate	
	n	Difference of means (95% CI)	Adjusted difference of means (95% CI)	Adjustments
T-helper_1_				
IFN-γ	88	0.061 (− 0.32, 0.44) P = 0.75	–	–
Tbet	89	− *0.40 (*− *0.76,* − *0.034)* *P *= *0.03*	− 0.21 (− 0.61, 0.18) P = 0.29	Age
T-helper_2_				
IL-13	89	− 0.74 (− 1.8, 0.36) P = 0.18	–	–
GATA3	89	− 0.12 (− 0.42, 0.18) P = 0.43	–	–
T-helper_17_				
IL-17	89	− 0.35 (− 0.80, 0.096) P = 0.12	− 0.087 (− 0.57, 0.40) P = 0.72	Age
RORC2	89	0.014 (− 0.35, 0.38) P = 0.94	–	–
T-regulatory				
IL-10	89	− 0.26 (− 0.61, 0.0871) P = 0.14	− 0.0070 (− 0.38, 0.36) P = 0.97	Age
IL-37	87	− *0.48 (*− *0.77,* − *0.19)* *P *= *0.001*	− *0.31 (*− *0.63,* − *0.0021)* *P *= *0.049*	Age
FOXP3	89	0.091 (− 0.31, 0.49) P = 0.65	–	–
TGF-β	89	− 0.0077 (− 0.32, 0.30) P = 0.96	–	–
Type I/III interferons				
IFN-α	87	− 0.41 (− 1.5, 0.7) P = 0.47	–	–
IFN-β	88	− 0.21 (− 1.0, 0.61) P = 0.62	–	–
IL-28	89	− 0.64 (− 1.5, 0.2) P = 0.13	–	–
IL-29	87	− 0.57 (− 1.5, 0.31) P = 0.15	–	–

Data are expressed as mean differences as a recurrently infected group minus hypertrophic group. The data were analysed using backward stepwise linear regression analysis after logarithmic transformation. Only significant co-factors were used as adjustments in the final model

CI, confidence interval; IFN, interferon; Tbet, T-box transcription factor; IL, interleukin; GATA3, GATA-binding factor 3; RORC, RAR-related orphan receptor C; FOXP, forkhead box protein; TGF, tumour growth factor

Significant values are shown in italic

## Discussion

This study shows differences in virus detections and T cell and interferon gene expressions in patients undergoing tonsillectomy due to tonsillar hypertrophy or recurrent tonsillitis. Patients with tonsillar hypertrophy were typically younger, and had more viral findings, but only bocavirus-1 was more often found in tonsils when compared to patients with recurrent tonsillitis. Respectively, they also had less self-reported pollen allergy, but no differences were found in food allergies between the groups. After age-adjusted analysis, tonsillar hypertrophy was associated with higher tonsillar mRNA expressions of IL-37. Other than age, no other significant co-factors were found.

IL-37 (formerly IL-1 family member 7) is a fundamental inhibitor of innate immunity [9, 10]. It has been shown to be expressed in macrophages, monocytes, plasma and epithelial cells [11]. After ligand activation, IL-37 inhibits inflammatory cytokines (especially IL-1β, but also IL-6, IL-7, IFN-γ, and TNF-α) and augments the level of anti-inflammatory IL-10 and T regulatory cells [11]. We have previously shown that the expression of IL-37 is closely and positively associated with other “immune activation/regulatory” cytokines (IL-10, IL-17, IL-37, TGF-β, FOXP3, GATA3, RORC2, Tbet) in tonsils [2]. The current analysis adds that tonsillar expression of anti-inflammatory cytokine IL-37 is also independently and positively associated with tonsillar hypertrophy.

Interferons (IFN-α, IFN-β, IFN-γ, IL-28, IL-29) are cytokines with antiviral activity and their expression is induced by viral infection. IL-28 and IL-29 are members of IFN-λ family [12, 13]. They are produced by dendritic cells and macrophages following viral infection or activation with bacterial components [12–14]. We expected to see differences in IFN expression (lower responses in recurrent tonsillitis than in tonsillar hypertophy group), since they have antiviral properties and they up-regulate the expression of MHC Class II molecules on cells which increases the immune system’s ability to recognize viruses [14, 15]. However, we did not observe these differences. We speculate that tonsillar hypertophy may be a consequence of chronic inflammation in tonsils and the same interferon pathways are equally activated in both conditions. We have previously found strong intragroup correlations of tonsillar IFN expression(IFN-α, IFN-β, IFN-γ, IL-28) [2].

Age was the main clinical characteristic differentiating the tonsillectomy indication groups. In agreement with previous findings [16], we found that obstruction due to the hypertrophy is more common with younger children where as adults have more recurrent tonsillitis. The age difference between the groups also explains the differences in smoking and in additional adenotomy performed. Interestingly, Reis and colleagues found no difference between the age distribution of hypertrophy and tonsillitis patients, but the narrow age range of their subjects (ages 2–11 years) may explain the lack of difference [17].

Virus was found in the nasopharynx of 79% of patients with tonsillar hypertrophy group and 38% in recurrent tonsillitis group. Most often detected viruses were adenovirus, bocavirus-1, coronavirus and rhinovirus. However, intratonsillar virus detection was low and did not show any statistically significant differences except for bocavirus-1. The results of nasopharyngeal and intratonsillar virus detection vs tonsillar cytokine responses are discussed in detail in our previous report [2].

Small differences in cytokine expression may partly be explained by differences concerning tonsillar germinal centers. The mean follicular area has been found to be larger, and the number of germinal centers higher, in the hypertrophy group compared to the recurrent tonsillitis [17–19]. In our study, the samples were taken from inside of the tonsils to minimize the margin of error and the possibility to misinterpreted differences between the groups. Seasonal changes, e.g. pollen and influenza seasons, may affect the expression of peripheral T cells [20], but we found no differences in tonsillar expression of cytokines between the seasons of the surgery. Also, respiratory viruses are continuously detected in children with chronic tonsillitis throughout the year [21–23]. Circulating serum 25(OH)D level has been shown to been positively associated with IL-37 level [3], but here it did not confound the results.

A limitation of the current study is that we did not investigate bacterial colonization of the tonsils in these patients due to fact that the operation was done during an afebrile period of their chronic tonsil condition. The downstream signaling of IL-37 is a complex process and to show functionality of IL-37 by downstream mediators was not in the scope of this study. In addition to forming cell-surface receptor complexes, IL-37 translocates to the nucleus where it binds to nuclear DNA and participate in transcription. [24, 25] IL-37 is regarded as a “dual function” cytokine, similar to IL-1α and IL-33.

## Conclusions

In summary, this study provides new insights about T cell research in lymphoid tissue from the clinical aspect of the surgical indication for tonsillectomy. We found tonsils as a good in vivo model for investigating the mechanisms of inflammatory processes and infections in lymphoid organs. Our data suggest that T-cell and interferon gene expressions appear to relatively stable over the two main indications of tonsillectomy, tonsillar hypertrophy and recurrent tonsillitis. However, anti-inflammatory immune responses, namely IL-37, might be slightly stronger in patients with tonsillar hypertrophy than with patients with recurrent tonsillitis.

## Additional file

**Additional file 1.** Health questionnaire.
