# Supplementary material for: Tonsillar cytokine expression between patients with tonsillar hypertrophy and recurrent tonsillitis
Source: Clin Transl Allergy. 2018 May 22;8:22. doi: 10.1186/s13601-018-0205-z (PMC5963068; doi:10.1186/s13601-018-0205-z)
Supplement: Supplementary file 1 — Additional file 1. Health questionnaire. [file 13601_2018_205_MOESM1_ESM.docx]

**SUPPLEMENT TABLE.** Health questionnaire.

**Date filled**: ___.___. 200___

Patient’s name _____________________________________________________________
Social security number _____________________________________________________________
Guardian’s name _____________________________________________________________
Address _____________________________________________________________
Phone _____________________________________________________________

**Operation day:** same as above no , what: ___.___. 200___
**Indication for the operation:** Recurrent/chronic otitis media infection yes no
 Recurrent tonsillitis yes no
 Periodic fever yes no
 Nasopharyngeal obstruction or apnea yes no
 Other, what? _______________________________________________
**Height:** _______ cm Weight: _______ cm

**Respiratory symptoms on the operation day:** yes no
If yes: Cough yes no
 Rhinitis yes no
 Acute otitis media yes no
 Pharyngitis yes no
 Other, what? _______________________________________________________________

**Respiratory symptoms within one month prior to the operation day:** yes no
If yes, when for the last time: Cough ____ days ago
 Rhinitis ____ days ago
 Acute otitis media ____ days ago
 Pharyngitis ____ days ago
 Expiratory breathing difficulty ____ days ago
Other, what? ___________________________________________ ____ days ago

**Medical record for the last 12 months (number of illness)**

Upper respiratory infection ____ times, acute otitis media ____ times, bronchitis ___ times, expiratory breathing difficulty ____ times, pneumonia ____ times, allergic rhinitis ___ times, exacerbation of atopic eczema ____ times, other illness, what _______________________________________________________, antibiot ____ times, systemic corticosteroid, ____ times, regular (>one month) medication, what __________________________________________________________________________________________________________________________________________________________________________________

**Allergic illnesses ever:**Any allergy yes no
 if yes, what/which ______________________________________________________________________
Allergic rhinitis yes no
Doctor-diagnosed atopic eczema yes no
Doctor-diagnosed asthma yes no

**Environment and family: Mother Father**Doctor-diagnosed asthma (ever) yes no yes no
Allergic rhinitis yes no yes no
Smoking yes no yes no
Smoking, self yes no
Furry pets yes no
Number of children in the household ____ children
Day-care: Home Family day-care Kindergarden/school
